# Only-Child Status in Relation to Perceived Stress and Studying-Related Life Satisfaction among University Students in China: A Comparison with International Students

**DOI:** 10.1371/journal.pone.0144947

**Published:** 2015-12-16

**Authors:** Janet Junqing Chu, Mobarak Hossain Khan, Heiko J. Jahn, Alexander Kraemer

**Affiliations:** Department of Public Health Medicine, School of Public Health, University of Bielefeld, P.O. Box 10 02 91, 33502 Bielefeld, Germany; Indiana University, UNITED STATES

## Abstract

**Objectives:**

University students in general face multiple challenges, which may affect their levels of perceived stress and life satisfaction. Chinese students currently face specific strains due to the One-Child Policy (OCP). The aim of this study was to assess (1) whether the levels of perceived stress and studying-related life satisfaction are associated with only-child (OC) status after controlling for demographic and socio-economic characteristics and (2) whether these associations differ between Chinese and international students.

**Materials and Methods:**

A cross-sectional health survey based on a self-administrated standardised questionnaire was conducted among 1,843 (1,543 Chinese, 300 international) students at two Chinese universities in 2010–2011. Cohen’s Perceived Stress Scale (PSS-14) and Stock and Kraemer’s Studying-related Life Satisfaction Scale were used to measure perceived stress and studying-related life satisfaction respectively. Multivariable logistic regression analyses were used to examine the associations of OC status with perceived stress and studying-related life satisfaction by sex for Chinese students and international students separately.

**Results:**

The Chinese non-only-children (NOCs) were more likely to come from small cities. Multivariable regression models indicate that the Chinese NOCs were more stressed than OCs (OR = 1.39, 1.11–1.74) with a stronger association in men (OR = 1.48, 1.08–2.02) than women (OR = 1.26, 0.89–1.77). NOCs were also more dissatisfied than their OC fellows in the Chinese subsample (OR = 1.37, 1.09–1.73). Among international students, no associations between OC status and perceived stress or studying-related life satisfaction were found.

**Conclusions:**

To promote equality between OCs and NOCs at Chinese universities, the causes of more stress and less studying-related life satisfaction among NOCs compared to OCs need further exploration.

## Introduction

University students in general are vulnerable to multiple stressors as a result of the challenges they face during their studies. These diverse challenges can include establishing self-reliance, maintaining a good level of academic achievement, and managing the basic demands of everyday life [1, 2]. Despite the fact that China houses more than one-fifth of the world’s college students [3, 4], the majority of research on stress and life satisfaction in university students has taken place in the USA and Europe [5–8]. Furthermore, the current generation of Chinese university students has to cope with a specific situation. They often do not have siblings due to the One-Child Policy (OCP) introduced in 1979 to reduce China’s population growth. The policy stipulates that each family can only have one child and it is strictly implemented, especially in urban areas among the Han ethnic group (around 91.5% of China’s total population) [9, 10]. A second birth is approved and permitted by the government under certain conditions, such as when urban families have disabled firstborns or are blended families in which one parent has no biological offspring. In rural areas, some families can have another child, depending on their location, firstborn’s sex, parental professions, and duration after the birth of the firstborn [10].

The OCP was implemented in conjunction with a set of administrative interventions such as residential registration, and a certificate of birth approval before becoming pregnant [10]. The proportion of only-children (OCs) in large cities jumped from about 8% of the birth cohort in 1973 to over 80% in 1985 [11]. Siblings can play a critical role in child socialization, such as learning to think from others’ perspectives, developing the ability to interact with peers, and practicing skills learned from others [12–14]. It has also been shown that siblings can act as buffers against stress in crisis situations [13]. Many psychologists in the West and in traditional China have perceived OCs as socially deprived, maladjusted, dependent and attention-seeking [15–17]. However, individuals growing up as OCs or non-only-children (NOCs) might have encountered different situations since the implementation of the OCP in China. The state-wide OCP guarantees a positive attitude toward only-child (OC) families. OC families enjoy privileges over other families with more than one child in terms of their children’s schooling and medical care or housing allocation etc [18]. In contrast, the Chinese society regards NOCs as less socially acceptable. These children generally grow up sensing such negative perceptions, and thus may feel more stressed and less satisfied with their lives [19]. Elevated stress levels and life dissatisfaction have both revealed associations with compromised physical and psychological health in young people [6–8]; as well as with poor academic achievement of university students [20]. Many factors at national, regional, local, and family levels influence life satisfaction [21]. The strengthening of students’ general life satisfaction is an important mission of education. In order to make institutional efforts to enhance the quality of life, well-being and academic development of the students, it is essential to identify factors associated with the life satisfaction of students in specific aspects regarding their studying experience and learning environment [22]. It has been reported that socio-economic status of the parents, financial freedom, lifestyle-related behaviours, social support, religious beliefs, self-esteem level, and OC status were associated with students’ life satisfaction and their level of perceived stress [13, 21–24].

Studies investigating whether and how OC status under the OCP in China is related to levels of perceived stress and studying-related life satisfaction among university students are still scarce. Moreover, findings regarding associations between perceived stress, life satisfaction and OC status in China are inconclusive. For instance, Liu and colleagues (2005) reported less perceived stress and better well-being among students with siblings than OCs [17]. In contrast, other studies have shown that OCs perceived better health and more life satisfaction than NOCs [19, 23]. While another study, however, found no differences between OCs and NOCs in this regard [25]. The inconsistent findings may be caused by the heterogeneous study design characteristics, study locations, and time of data collection. Liu et al (2005), for instance, found discrepancy between rural and urban areas in terms of the difference between OCs and NOCs: urban OCs perceived more stress than NOCs, while no difference found in perceived stress between rural OCs and NOCs [17]. Deviating findings may also result from the method of data collection. In the Jiao et al [16] study the teachers made the evaluation concerning the wellbeing of the students. In the Falbo et al (1989) study mothers provided the collected information on students’ psychological wellbeing and satisfaction [25]. Additionally, the studies cited were conducted during different time periods, from relatively shortly after the OCP was implemented (Jiao et al 1986) to more recent studies conducted in 2009 [23] and 2013 [19]. Taking into account the fundamental social and economic changes in China, it is plausible that this may have had an effect on the different study results.

Our study has included subjects from universities in different locations that recruit urban and rural students from all over China. The survey was completed within a year using a self-administrated standardised questionnaire that was applied for the Cross National Student Health Study conducted in Europe. Self-rating of health is a feasible way to measure health in large-scale surveys, and it has been shown to have high reliability, validity and predictive power for measuring objective and subjective health in adult population groups [2]. Furthermore, to our knowledge, no study has reported whether and how the relations between OC status, perceived stress and studying related life satisfaction among Chinese students differ from international students. Based on the abovementioned background, the main objectives of our study were to assess: (1) whether the levels of perceived stress and studying-related life satisfaction are associated with the OC status after controlling for demographic, socio-economic characteristics and (2) whether these associations differ between Chinese and international students. Through this approach, our study may provide insights helping both academics and policy makers to understand the characteristics of the perceived stress and studying-related life satisfaction among students at Chinese universities in the present era.

## Materials and Methods

### Study location and questionnaire

The data were obtained from a survey administered in 2010–2011 at two Chinese universities—Sun Yat-sen University (SYSU) in Guangzhou and Peking University (PKU) in Beijing. The questionnaire used in the survey was an adjusted version of the standardised questionnaire for the Cross National Student Health Study conducted in seven European countries from 1998 to 2005. At both universities, one-third of the student sample was from the medical sciences, one-third from other health sciences (dentistry, nursing, public health and pharmacy), and the rest from the natural sciences and economics, with approximately half the sample coming from each university. The response rate was above 90% at both universities. Altogether 1,853 undergraduate students completed the questionnaire. Ten of them did not state whether they were Chinese or international students. Among the 300 international students, 41.3% were from South Korea, 25.7% were from Taiwan, 7.7% were from India. Nine international students did not state their country of origin, while the rest of 67 students were from North America, Mideast, Africa and other Asian countries.

Ethical approval for the study was obtained from the Institutional Review Board of Peking University. The questionnaire was initially developed in English. Prior to the survey, the standardised questionnaire was slightly modified to adapt it to the Chinese situation (such as, adding an item asking whether the student is an OC) and afterwards translated by two researchers from English into Chinese. Two bilingual doctoral students examined the translated instruments. The two translated instruments were very close in meaning, indicating correct language transference. A pilot test was administered to a group of 32 undergraduate students (including three international students) at PKU. Since all international students took classes and exams in Chinese and had therefore no difficulties understanding and answering the Chinese questionnaire. Students were asked towards the end of lectures to complete the surveys. They were informed in written form that participation was voluntary and anonymous and that questionnaire completion was considered as study participation agreement. No participation incentives were provided.

### Measures

#### Perceived stress

Perceived stress was assessed by the 14-item Perceived Stress Scale (PSS-14), which measures the degree to which a respondent appraises situations in his or her life during the previous four weeks as stressful [26]. The PSS-14 has been translated into 25 languages and used in different countries; it is an easy to use scale with acceptable test-retest reliability, criterion validity, and known-group validity [27]. These 14 items used a 5-point Likert-type scale response format, ranging from 0 = “never” to 4 = “very often”. PSS-14 scores were obtained by summing across all 14 items. The scale yielded a single score, with higher scores indicating higher levels of stress ranging from 0 to 56. Cronbach’s alpha of the PSS-14 scale in our sample was 0.79.

#### Studying-related life satisfaction

Studying-related life satisfaction was measured by the 18-item Studying-related Life Satisfaction Scale from Stock and Kraemer (2001) with the question “To what extent are you satisfied with the following aspects of your life” (6-point Likert-type scale from 1 = “very unsatisfied” to 6 = “very satisfied”). Questions concerned the following topics: Studies in general, major and minor subjects, grades at university, social integration at university, job opportunities, accommodation, neighbourhood, place of study, free time, financial situation, relations with friends, relations with parents, private life, health, China as the country of study, political and economic situation in China, and life in general [28]. The studying-related life satisfaction scale has been developed in Germany and used in German as well as in other European university student health surveys with acceptable reliability and validity [28–30]. The sum score of these 18 items produced a possible range from 18 to 108 with higher scores indicating higher levels of life satisfaction. In our sample Cronbach’s alpha of the studying related life satisfaction scale was 0.91.

#### Demographic, socio-economic and lifestyle-related characteristics

Twelve variables were included to assess participants’ demographic, socio-economic and lifestyle-related characteristics: sex, age, birthplace (“countryside”/“small city”/“large city”), OC status (“yes”/”no”), mother’s education (“college or above” [recoded as “high”]/“lower than college level” [recoded as “low”]), father’s education (“college or above” [recoded as “high”]/“lower than college level” [recodes as “low”]), international student status (“yes”/”no”), respondent’s self-rated economic situation—how sufficient students considered the amount of money they have at their disposal (“sufficient”/“insufficient”), satisfaction with social support received in crisis situations such as feeling depressed or stressed (“satisfied”/“unsatisfied”), religious beliefs (“yes”/”no”), studying university (PKU/SYSU) and the frequency of physical activity (“less than once a week”/“1–2 times a week”/“at least 3 times a week”).

### Statistical analysis

Frequencies and medians were tabulated for descriptive analysis. Mann-Whitney-U tests (for continuous variables) and Pearson’s Chi-square tests (for categorical variables) were used for between group (Chinese vs. international, OC vs. NOC) comparison in demographic, socio-economic and lifestyle-related variables. PSS-14 and studying-related life satisfaction scale were compared between OC and NOC students using Mann-Whitney-U tests. The total sum scores of perceived stress and studying-related life satisfaction scales were dichotomized based on their median, yielding students stressed (> 25) vs. students less stressed (≤ 25), and students dissatisfied (≤ 73) vs. students satisfied (> 73) respectively, for further analyses. Sum score median as cut-off value for logistic regression analyses was used by previous studies for PSS-14 and life satisfaction scale in specific areas [31, 32]. We used bivariable logistic regression analyses to assess crude associations between demographic, socio-economic, and lifestyle-related variables with perceived stress and studying-related life satisfaction. For assessing the associations between OC status, perceived stress and studying-related life satisfaction, we included sex, age, father’s education, mother’s education, birth place, religious beliefs, self-rated economic situation, and studying university in the multivariable logistic regression models for statistical adjustment. All analyses were performed with SPSS^®^ for Windows version 21. The statistical tests were two-tailed, for all tests, the significance level was set at 0.05.

## Results

### Description of the sample

The main characteristics of the study population are presented in Table 1. International students accounted for 16.3% of the whole sample. Significant differences were observed in the following aspects between Chinese and international students: Chinese students were slightly younger, more satisfied with social support received in crisis situations and performed less physical activity than international students. The proportions of NOCs, students born in large cities, having religious beliefs and parents with a high level of education were higher among the international group. However, despite these partly strong differences, Chinese and international students did not differ with respect to perceived stress or studying-related life satisfaction (Table 1).

**Table 1:** Demographic, socio-economic and lifestyle-related characteristics of the sample.

Categorical variables	N (%)			^‡^p-value
	All subjects 1,843 (100)	Chinese 1,543 (83.7)	International 300 (16.3)	
Sex				
Male	948 (52.1)	804 (53.0)	142 (47.3)	0.071
Female	873 (47.9)	712 (47.0)	158 (52.7)	
Physical activity				
< 1 a week	510 (28.5)	442 (29.5)	67 (23.4)	***0*.*007***
1–2 a week	872 (48.8)	733 (49.0)	135 (47.2)	
≥ 3 a week	406 (22.7)	321 (21.5)	84 (29.4)	
Father’s education				
High	810 (45.8)	596 (39.8)	213 (79.2)	***< 0*.*001***
Low	958 (54.2)	900 (60.2)	56 (20.8)	
Mother’s education				
High	680 (38.2)	493 (32.8)	186 (67.1)	***< 0*.*001***
Low	1100 (61.8)	1008 (67.2)	91 (32.9)	
Only-child				
Yes	1092 (60.0)	999 (65.9)	92 (30.8)	***< 0*.*001***
No	727 (40.0)	516 (34.1)	207 (69.2)	
Birth place				
Countryside	596 (32.8)	569 (37.5)	26 (8.8)	***< 0*.*001***
Small city	623 (34.3)	556 (36.7)	65 (22.0)	
^†^Large city	598 (32.9)	392 (25.8)	205 (69.2)	
Social support				
Satisfied	1336 (73.0)	1166 (76.3)	164 (56.0)	***< 0*.*001***
Unsatisfied	494 (27.0)	362 (23.7)	129 (44.0)	
Self-rated economic situation				
Sufficient	1466 (81.2)	1220 (81.2)	241 (81.1)	0.992
Insufficient	339 (18.8)	283 (18.8)	56 (18.9)	
Religious beliefs				
Yes	297 (16.3)	131 (8.6)	165 (55.7)	***< 0*.*001***
No	1521 (83.7)	1386 (91.4)	131 (44.3)	
**Continuous variables**	**Median (quartile deviation)**			
Perceived stress scale scores	25.0 (5.0)	25.0 (5.5)	26.0 (3.5)	0.159
Studying-related life satisfaction scale scores	72.0 (9.0)	72.0 (9.0)	72.0 (9.6)	0.683
Age	21.0 (1.5)	21.0 (1.5)	22.0 (1.8)	***< 0*.*001***

^†^: Cities higher than county level

^‡^: Chi-square test for categorical variables, Mann-Whitney-U test for continuous variables

### Characteristics of the sample by only-child status

Characteristics of the Chinese and international students by OC status are presented in Table 2. Among the Chinese, NOC status was associated with born in a small city. Among the international students, NOC status was associated with being born in a large city, having more physical activity, and having a father with high education degree. Interestingly, the OC status did not seem to be associated with social support received in crisis situations among the Chinese students whereas the international NOCs reported significantly more frequent to be satisfied with social support received in crisis situations as compared to international OCs (Table 2).

**Table 2:** Characteristics of Chinese and international students by only-child status.

variables	Chinese N (%)			International N (%)		
	Only-child 999 (65.9)	Non-only-child 516 (34.1)	^‡^p-value	Only-child 92 (30.8)	Non-only-child 207 (69.2)	^‡^p-value
Sex						
Male	514 (51.6)	288 (55.9)	0.107	38 (41.3)	104 (50.2)	0.153
Female	483 (48.4)	227 (44.1)		54 (58.7)	103 (49.8)	
Physical activity						
< 1 a week	284 (29.0)	154 (30.5)	0.799	30 (35.7)	37 (18.4)	***0*.*002***
1–2 a week	486 (49.5)	242 (47.9)		39 (46.4)	96 (47.8)	
≥ 3 a week	211 (21.5)	109 (21.6)		15 (17.9)	68 (33.8)	
Father’s education						
High	387 (39.2)	207 (41.1)	0.478	51 (68.9)	161 (83.0)	***0*.*011***
Low	601 (60.8)	297 (58.9)		23 (31.1)	33 (17.0)	
Mother’s education						
High	337 (34.0)	154 (30.4)	0.153	49 (62.0)	136 (69.0)	0.263
Low	653 (66.0)	353 (69.6)		30 (38.0)	61 (31.0)	
Birth place						
Countryside	395 (39.6)	173 (33.6)	***< 0*.*001***	17 (19.1)	9 (4.4)	***< 0*.*001***
Small city	330 (33.0)	224 (43.5)		28 (31.5)	37 (18.0)	
^†^Large city	273 (27.4)	118 (22.9)		44 (49.4)	160 (77.6)	
Social support						
Satisfied	756 (76.1)	394 (77.0)	0.698	37 (43.0)	126 (61.2)	***0*.*004***
Unsatisfied	238 (23.9)	118 (23.0)		49 (57.0)	80 (38.8)	
Self-rated economic situation						
Sufficient	792 (80.1)	425 (83.3)	0.127	76 (83.5)	164 (80.0)	0.476
Insufficient	197 (19.9)	85 (16.7)		15 (16.5)	41 (20.0)	
Religious beliefs						
Yes	85 (8.5)	45 (8.7)	0.885	65 (72.2)	105 (51.2)	***< 0*.*001***
No	913 (91.5)	470 (91.3)		25 (27.8)	100 (48.8)	
	**Median (quartile deviation)**			**Median (quartile deviation)**		
Age	21.0 (1.5)	21.0 (1.5)	0.197	23.0 (1.5)	21.0 (1.5)	***< 0*.*001***

^†^: Cities higher than county level

^‡^: Chi-square test for categorical variables, Mann-Whitney-U test for continuous variables

### Associations between only-child status, items of PSS-14 and studying-related life satisfaction scale

Associations between only-child status, items of perceived stress scale and studying-related life satisfaction scale are presented in Table 3. Among Chinese, NOCs rated higher score than OCs on all items of perceived stress scale though not statistically significant (apart from the item of being able to spend time freely). NOCs scored lower than OCs on all items of the studying-related life satisfaction scale with statistical significance in the following aspects: studies in general, grades at university, integration at university, job opportunities, relations with friends, and political situation in China. While in the international group, for both scales NOCs rated mixed results, higher on some items, and lower on other items compared with their OC peers.

**Table 3:** Associations between only-child status (OC vs. NOC), and items of studying-related life satisfaction scale and perceived stress scale.

Studying related life satisfaction scale item	(^†^Median, quartile deviation) and Mann-Whitney-U-test p-value				Perceived stress scale item	(^‡^Median, quartile deviation) and Mann-Whitney-U-test p-value			
	Chinese		International			Chinese		International	
Studies in general	OC (4.0, 1.0)	***0*.*001***	OC (4.0, 1.0)	0.083	-been upset because something unexpected happened in your life	OC (2.0, 1.0)	0.615	OC (2.0, 1.0)	0.526
	NOC (4.0, 0.5)		NOC (4.0, 0.5)			NOC (2.0, 1.0)		NOC (2.0, 1.0)	
Major and minor/subjects	OC (4.0, 1.0)	0.218	OC (4.0, 1.0)	0.297	-had the impression that the most important things in your life are out of your control	OC (1.0, 1.0)	0.445	OC (2.0, 1.0)	***0*.*015***
	NOC (4.0, 1.0)		NOC (4.0, 0.5)			NOC (1.0, 1.0)		NOC (1.0, 0.5)	
Grades at university	OC (4.0, 0.5)	***0*.*019***	OC (4.0, 0.5)	0.176	- felt nervous and tense	OC (2.0, 0.5)	0.440	OC (2.0, 1.0)	0.077
	NOC (3.0, 0.5)		NOC (3.0, 0.5)			NOC (2.0, 1.0)		NOC (1.0, 0.5)	
Integration at university	OC (4.0, 1.0)	***0*.*001***	OC (4.0, 1.0)	0.557	^±^- succeeded in dealing with unpleasant events	OC (2.0, 0.5)	0.062	OC (2.0, 0.5)	***0*.*013***
	NOC (4.0, 1.0)		NOC (4.0, 1.0)			NOC (2.0, 1.0)		NOC (2.0, 0.6)	
Job opportunities	OC (4.0, 1.0)	***0*.*004***	OC (3.0, 1.0)	0.815	^±^-had an impression that you were able to deal with important changes in your life	OC (1.0, 0.5)	0.060	OC (1.0, 0.5)	0.161
	NOC (4.0, 0.5)		NOC (3.0, 1.0)			NOC (1.0, 0.5)		NOC (2.0, 0.5)	
Accommodation	OC (4.0, 1.0)	0.198	OC (4.0, 1.0)	***0*.*026***	^±^- felt sure to be able to deal with your personal problems well enough	OC (1.0, 1.0)	0.156	OC (1.0, 0.5)	0.821
	NOC (3.0, 1.0)		NOC (4.0, 1.0)			NOC (1.0, 0.5)		NOC (1.0, 0.5)	
Neighbourhood	OC (5.0, 0.5)	0.255	OC (4.0, 1.0)	***< 0*.*001***	^±^- had an impression that things in your life developed as you planned	OC (2.0, 0.5)	0.077	OC (2.0, 1.0)	0.418
	NOC (5.0, 0.5)		NOC (4.0, 1.0)			NOC (2.0, 1.0)		NOC (2.0, 0.6)	
Place of study	OC (5.0, 0.5)	0.491	OC (4.0, 1.0)	0.316	- had an impression that you did not meet everyday demands	OC (1.0, 0.5)	0.869	OC (1.0, 0.5)	0.395
	NOC (4.5, 0.5)		NOC (4.0, 1.0)			NOC (1.0, 0.5)		NOC (1.0, 0.5)	
Free time	OC (4.0, 1.0)	0.226	OC (4.0, 1.0)	0.708	^±^- succeeded in getting rid of vexations/OR nuisances from your way	OC (1.0, 0.5)	0.157	OC (2.0, 1.0)	0.853
	NOC (4.0, 1.0)		NOC (4.0, 1.0)			NOC (2.0, 0.5)		NOC (2.0, 1.0)	
Financial situation	OC (4.0, 1.0)	0.204	OC (4.0, 1.0)	0.902	^±^- had an impression that you were at the top	OC (3.0, 0.5)	0.574	OC (2.0, 1.0)	***0*.*005***
	NOC (4.0, 1.0)		NOC (4.0, 1.0)			NOC (3.0, 0.5)		NOC (2.0, 0.5)	
Relations with friends	OC (5.0, 0.5)	***0*.*013***	OC (4.0, 1.0)	0.186	- felt angry that things happened which were out of your control	OC (2.0, 1.0)	0.319	OC (2.0, 1.0)	***0*.*012***
	NOC (5.0, 0.5)		NOC (5.0, 0.5)			NOC (2.0, 1.0)		NOC (2.0, 1.0)	
Relation with parents	OC (5.0, 0.5)	0.266	OC (5.0, 1.0)	0.605	- thought about things which you had to complete	OC (3.0, 1.0)	0.116	OC (2.0, 0.5)	0.619
	NOC (5.0, 1.0)		NOC (5.0, 1.0)			NOC (3.0, 1.0)		NOC (3.0, 0.5)	
Private life	OC (5.0, 0.5)	0.228	OC (5.0, 1.0)	0.532	^±^- felt you were able to spend your time freely	OC (2.0, 1.0)	***0*.*019***	OC (2.0, 1.0)	0.763
	NOC (5.0, 0.5)		NOC (4.5, 1.0)			NOC (2.0, 1.0)		NOC (2.0, 1.0)	
Health	OC (4.0, 0.5)	0.123	OC (4.0, 0.5)	0.659	-had an impression that difficulties overwhelmed you so much that you were not able to accomplish them	OC (1.0, 0.5)	0.557	OC (2.0, 0.5)	0.062
	NOC (4.0, 1.0)		NOC (4.0, 1.0)			NOC (1.0, 0.5)		NOC (1.0, 0.5)	
Chinas as the study country	OC (4.0, 1.0)	0.158	OC (4.0, 1.0)	0.768					
	NOC (4.0, 1.0)		NOC (4.0, 1.0)						
Political situation in China	OC (4.0, 1.0)	***0*.*017***	OC (4.0, 1.0)	***< 0*.*001***					
	NOC (4.0, 0.5)		NOC (3.0, 0.5)						
Economic situation in China	OC (4.0, 1.0)	0.372	OC (5.0, 1.0)	***< 0*.*001***					
	NOC (4.0, 1.0)		NOC (4.0, 0.5)						
Satisfaction with life in general	OC (4.0, 0.5)	0.090	OC (5.0, 0.5)	0.108					
	NOC (4.0, 0.5)		NOC (4.0, 1.0)						

^†^: Answers selected from “very unsatisfied = 1” to “very satisfied = 6” for the question: “To what extent are you satisfied with the following areas of your life?”

^‡^: Answers selected from “never = 0” to “very often = 4” for the question: “In the course of the last four weeks, how often have you…”

^±^: revised item

### Crude associations between perceived stress, studying-related life satisfaction and relevant variables

Crude associations between demographic, socio-economic and lifestyle-related variables and PSS-14 scores (stressed vs. less stressed) and studying-related life satisfaction scale scores (dissatisfied vs. satisfied) are presented in Table 4. Amongst both Chinese and international students, self-rated economic situation as well as social support received in crisis situations revealed negative associations with perceived stress and positive associations with studying-related life satisfaction. Among the Chinese students, being an OC was associated with being less stressed and more satisfied with studying-related life satisfaction. Less physical activity and having religious beliefs were associated with being more stressed. In both Chinese and international students no gender difference was found in perceived stress and studying related life satisfaction.

**Table 4:** Crude associations of demographic, socio-economic and lifestyle-related factors with perceived stress (stressed vs. less stressed) and studying-related life satisfaction (dissatisfied vs. satisfied).

Categorical variables	Odds ratio (OR) and 95% confidence interval (95% CI)			
	High Perceived Stress		Studying-Related Life Dissatisfaction	
	Chinese	International	Chinese	International
**Sex**				
Male (^†^Ref.)				
Female	1.07 (0.87–1.31)	1.11 (0.69–1.79)	1.10 (0.89–1.35)	1.14 (0.70–1.84)
**Father’s education**				
High (Ref.)				
Low	1.16 (0.93–1.44)	0.73 (0.43–1.25)	1.19 (0.96–1.49)	1.44 (0.84–2.46)
**Mother’s education**				
High (Ref.)				
Low	1.15 (0.93–1.42)	1.31 (0.69–2.51)	1.15 (0.93–1.42)	1.65 (0.87–3.12)
**Birth place**				
Countryside (Ref.)				
Small city	1.14 (0.90–1.44)	0.59 (0.20–1.76)	1.14 (0.89–1.44)	0.89 (0.33–2.40)
^‡^Large city	0.90 (0.69–1.17)	0.41 (0.15–1.11)	0.87 (0.66–1.13)	0.79 (0.32–1.93)
**Physical activity**				
≥ 3 a week (Ref.)				
1–2 a week	1.28 (0.97–1.68)	1.57 (0.89–2.78)	**1.39** * **(1.06–1.82)**	1.28 (0.72–2.26)
< 1 a week	**1.97** *** **(1.46–2.65)**	1.74 (0.89–3.43)	**2.03** *** **(1.51–2.74)**	1.16 (0.59–2.30)
**Religious beliefs**				
No (Ref.)				
Yes	**1.46** * **(1.02–2.09)**	1.41 (0.87–2.28)	0.94 (0.65–1.35)	0.81 (0.50–1.32)
**Self-rated economic situation**				
Sufficient (Ref.)				
Insufficient	**1.51** ** **(1.15–1.96)**	**2.00** * **(1.06–3.79)**	**2.28** *** **(1.72–3.01)**	**2.67** ** **(1.34–5.32)**
**Social support**				
Sufficient (Ref.)				
Insufficient	**3.43** *** **(2.65–4.43)**	**2.50** *** **(1.52–4.10)**	**3.35** *** **(2.56–4.38)**	**1.74** * **(1.06–2.86)**
**University**				
Peking University (Ref.)				
Sun Yat-sen University	1.20 (0.98–1.48)	**2.33** ** **(1.31–4.15)**	**1.71** *** **(1.39–2.10)**	0.77 (0.44–1.34)
**Only-child**				
Yes (Ref.)				
No	**1.38** ** **(1.11–1.71)**	0.63 (0.37–1.08)	**1.36** ** **(1.09–1.69)**	1.13 (0.66–1.95)
**Age**	1.01 (0.96–1.06)	1.06 (0.95–1.17)	**1.08** ** **(1.02–1.13)**	1.01 (0.91–1.12)

^†^: Reference category

^‡^: Cities higher than county level

Significance of Wald test:

*p < 0.05

**p < 0.01

***p < 0.001

### Adjusted associations between only-child status, perceived stress and studying-related life satisfaction

The associations between OC status, perceived stress (stressed vs. less stressed), and studying-related life satisfaction (dissatisfied vs. satisfied) are presented in Table 5 (without stratification by sex) and Table 6 (stratified by sex). In these multivariable analyses, results were adjusted by sex, age, father’s education, mother’s education, birth place, religious beliefs, self-rated economic situation, and studying university. Chinese NOCs were more likely to report being stressed and dissatisfied compared with their OC peers (Table 5). According to sex-stratified analysis, the association between being stressed and NOC status remained significant only for Chinese men (Table 6).Amongst the international students, OC status was insignificantly associated with being stressed or dissatisfied with studying-related life satisfaction (Tables 5 and 6).

**Table 5:** Adjusted odds ratio (OR) and 95% confidence interval (95% CI) for only-child status, perceived stress (stressed vs. less-stressed), and studying-related life satisfaction (dissatisfied vs. satisfied).

Variables	OR (95% CI)			
	High Perceived Stress		Studying-Related Life Dissatisfaction	
	Chinese ^†^R^2^ = 0.03 (N = 1,409, p = 0.003)	International R^2^ = 0.06 (N = 231, p = 0.370)	Chinese R^2^ = 0.06 (N = 1,393, p < 0.001)	International R^2^ = 0.08 (N = 229, p = 0.210)
**Sex**				
Male (^‡^Ref.)				
Female	1.12 (0.90–1.38)	1.13 (0.66–1.93)	1.24 (1.00–1.55)	1.12 (0.65–1.92)
**Father’s education**				
High (Ref.)				
Low	0.97 (0.70–1.35)	**0.46** * **(0.22–0.98)**	0.99 (0.71–1.38)	1.24 (0.59–2.63)
**Mother’s education**				
High (Ref.)				
Low	1.12 (0.82–1.53)	1.95 (0.77–4.93)	0.98 (0.71–1.35)	1.23 (0.49–3.07)
**Birth place**				
Countryside (Ref.)				
Small city	1.13 (0.86–1.49)	1.24 (0.34–4.51)	1.13 (0.86–1.50)	0.85 (0.24–2.99)
^¶^Large city	0.98 (0.71–1.37)	1.07 (0.32–3.65)	1.07 (0.76–1.49)	0.66 (0.20–2.17)
**Religious beliefs**				
No (Ref.)				
Yes	1.37 (0.94–2.00)	1.38 (0.79–2.42)	0.81 (0.46–1.43)	0.81 (0.46–1.43)
**Self-rated economic situation**				
Sufficient (Ref.)				
Insufficient	**1.60** ** **(1.21–2.13)**	1.72 (0.82–3.61)	**2.31** *** **(1.71–3.11)**	**2.88** * **(1.29–6.47)**
**University**				
Peking University (Ref.)				
Sun Yat-sen University	1.19 (0.87–1.62)	1.67 (0.60–4.64)	**1.77** *** **(1.30–2.42)**	0.51 (0.17–1.53)
**Age per year increase**	0.95 (0.88–1.02)	1.03 (0.91–1.18)	0.95 (0.88–1.03)	1.04 (0.91–1.18)
**Only-child**				
Yes (Ref.)				
No	**1.39** ** **(1.11–1.74)**	1.13 (0.44–2.87)	**1.37** ** **(1.09–1.73)**	0.78 (0.28–2.13)

^†^: In all regression models Nagelkerke R^2^ was reported, df was 10

^‡^: Reference category

^¶^: Cities higher than county level

Significance of Wald test:

*p < 0.05

**p < 0.01

***p < 0.001

**Table 6:** Adjusted odds ratio (OR) and 95% confidence interval (95% CI) for only-child status, perceived stress (stressed vs. less-stressed), and studying-related life satisfaction (dissatisfied vs. satisfied) by sex.

Variables	OR (95% CI)							
	High Perceived Stress				Studying-Related Life Dissatisfaction			
	Chinese		International		Chinese		International	
	Male ^†^R^2^ = 0.03 (N = 742, p = 0.040)	Female R^2^ = 0.04 (N = 667, p = 0.015)	Male R^2^ = 0.08 (N = 113, P = 0.640)	Female R^2^ = 0.17 (N = 118, P = 0.057)	Male R^2^ = 0.04 (N = 732, P = 0.003)	Female R^2^ = 0.10 (N = 661, P < 0.001)	Male R^2^ = 0.09 (N = 118, P = 0.550)	Female R^2^ = 0.12 (N = 111, P = 0300)
**Father’s education**								
High (^‡^Ref.)								
Low	0.69 (0.43–1.10)	1.45 (0.91–2.32)	0.79 (0.28–2.24)	**0.23** * **(0.07–0.74)**	0.92 (0.57–1.47)	1.07 (0.67–1.73)	1.21 (0.43–3.37)	1.48 (0.45–4.89)
**Mother’s education**								
High (Ref.)								
Low	1.35 (0.85–2.14)	0.94 (0.60–1.46)	1.04 (0.29–3.77)	3.97 (0.91–17.21)	0.99 (0.62–1.58)	0.96 (0.61–1.51)	1.06 (0.30–3.73)	1.37 (0.33–5.69)
**Birth place**								
Countryside (Ref.)								
Small city	1.08 (0.75–1.57)	1.18 (0.78–1.77)	1.64 (0.25–10.89)	0.78 (0.11–5.48)	1.27 (0.87–1.84)	1.00 (0.65–1.53)	1.02 (0.17–6.06)	0.82 (0.13–5.17)
^¶^Large city	0.85 (0.53–1.35)	1.11 (0.69–1.80)	1.05 (0.18–6.26)	0.87 (1.14–5.34)	1.34 (0.85–2.13)	0.85 (0.52–1.39)	0.52 (0.10–2.81)	0.85 (0.16–4.62)
**Religious beliefs**								
No (Ref.)								
Yes	1.20 (0.72–1.98)	1.70 (0.95–3.04)	1.22 (0.54–2.74)	1.92 (0.82–4.51)	0.94 (0.56–1.57)	0.97 (0.54–1.77)	1.15 (0.51–2.57)	0.67 (0.29–1.55)
**Self-rated economic situation**								
Sufficient (Ref.)								
Insufficient	**1.51** * **(1.04–2.19)**	**1.71** * **(1.10–2.66)**	0.58 (0.18–1.93)	**4.04** * **(1.33–12.26)**	**2.13** *** **(1.44–3.13)**	**2.50** *** **(1.55–4.02)**	2.58 (0.79–8.39)	3.21 (1.00–10.31)
**University**								
Peking University (Ref.)								
Sun Yat-sen University	1.09 (0.73–1.63)	1.36 (0.82–2.25)	3.23 (0.58–17.83)	0.93 (0.22–3.94)	1.31 (0.87–1.95)	**2.69** *** **(1.61–4.48)**	0.54 (0.11–2.58)	0.47 (0.09–2.49)
**Age per year increase**	1.03 (0.93–1.14)	**0.87** * **(0.77–0.97)**	0.99 (0.83–1.17)	1.14 (0.92–1.42)	1.01 (0.91–1.12)	**0.87** * **(0.78–0.98)**	1.09 (0.92–1.29)	0.97 (0.78–1.21)
**Only-child**								
Yes (Ref.)								
No	**1.48** * **(1.08–2.02)**	1.26 (0.89–1.77)	1.12 (0.23–5.36)	1.25 (0.35–4.47)	1.26 (0.92–1.72)	1.38 (0.97–1.97)	1.89 (0.43–8.39)	0.39 (0.09–1.75)

^†^: In all regression models Nagelkerke R^2^ was reported, df was 9

^‡^: Reference category

^¶^: Cities higher than county level

Significance of Wald test:

*p < 0.05

***p < 0.001

## Discussion

### Main findings

In this study we examined the associations between OC status and perceived stress, as well as the association between OC status and studying-related life satisfaction among university students in China. We found that Chinese NOCs were less satisfied than OCs in studying-related life satisfaction. The Chinese NOCs were also more stressed than OCs with a stronger association among men than women. Among the international students no association between OC status, perceived stress and studying-related life satisfaction was found.

### What is already known and what this study adds

Based on literature review, there are two major explanatory mechanisms to explain the advantages of OCs over NOCs: Firstly, the mechanism of “uniqueness” of the OCs, i.e. they have their parents’ undivided attention like the first borns, and they are also like the last borns who are never dethroned by the birth of a subsequent child [33]. Secondly, the mechanism of socio-economic advantages of the parents of OCs and the more intensive parent-child relationship provide advantages to OCs over NOCs [34, 35]. The advantages of the Chinese OCs over NOCs in perceived stress and studying-related life satisfaction may be to a certain extent explained by the following:

Firstly, in accepting the OCP, Chinese parents would provide more motivations for their OC to learn and also dedicate more energy and money to the future of the child. Because of the resulting higher academic achievements, teachers may take a positive view towards the OCs [36]. Our findings that Chinese OCs were more satisfied in aspects of “studies in general”, and “grades at university” lend support to these previous results.

Secondly, the OCP projected positive social perceptions towards OCs, while children with siblings, portrayed as “bad” by society, experience humiliations relative to their OC peers during their childhoods [19, 37]. It has been shown that perceived discrimination is negatively associated with life satisfaction and self-esteem in adolescents [38]. Low self-esteem, in turn, was observed to be related to increased stress and decreased life satisfaction in young people [7, 21]. Our results that Chinese NOCs were statistically less satisfied in “relations with friends” and “integration at university” than OCs, may give some hints in favour of OC. The OC status of the students is manifested at university in China. Firstly, due to the fact that one 20 m^2^ dormitory room is shared by 6–8 bachelor students [39], it is difficult to keep privacy among peers. Secondly, in order to ease the implementation of the OCP, some policy-related benefits such as financial aid for medical problems are only available to OCs by owning the OC certificate [36].

Finally, owing to the attributes of the OCP, NOC families exhibit some unusual characteristics: they represent ethnic minorities and have disabled children, etc. These features may also have an impact on NOCs’ perceptions of stress and studying-related life satisfaction.

Therefore, we argue that the parent-child relationship and the influence of the OCP may be the explanatory mechanisms for our findings that Chinese OCs seem to be less stressed and more satisfied than their NOC peers.

We also found a higher proportion of OCs amongst students born in the countryside than those born in cities (70% vs. 64%). This may imply a rural-urban educational inequality in China, which was also reported by Qian and Smyth (2008) [40]. It is worth remembering here that the OCP has been more strictly implemented in cities than in the countryside. This means that NOCs born in the countryside have less opportunity to study at university.

Regarding our findings of the gender difference among NOCs on perceived stress, son preference is well known in Chinese culture, but its intensity varies with social, economic, and cultural circumstances [41, 42]. It has been reported that son preference was more imperative among the families in rural areas, towns and small cities, particularly when the family’s firstborn was a female [42]. This phenomenon is understandable since the root causes for preferring boys such as helping in farming and providing financial security for parents in their old age are more relevant in these areas compared with in large cities, where the majority have insurance and pension. These cultural values and preference may put a great deal of pressure on men, especially those from rural and small towns. Due to the attributes of the OCP (in rural areas and small cities, couples may have the second child when their first child is a girl), NOC men are more likely from countryside or small cities with an elder sister as sibling. Our results that NOC status was associated with born in a small city are in agreement with this policy impact although we did not collect the information concerning birth order.

Our results confirmed previous findings that social support, financial freedom and physical activity are negatively associated with stress, and positively associated with life satisfaction in university students [43, 44]. These findings provide suggestions for intervention programs in reducing stress and increasing studying-related life satisfaction in Chinese university setting. For instance, promoting interpersonal interaction, facilitating sport at campus may help the Chinese NOCs integrating at university and improving their relations with peers.

### Strengths and limitations

The strengths of this study include the relatively large sample size, high response rate, and the consideration of international students as comparison group in the study. The majority of the international students were from South Korea and Taiwan–which have similar cultural traditions to Mainland China. This helped in reducing some cultural bias, and hence made the situation of Chinese and international students in a way comparable. We chose two large comprehensive universities (one from southern China and one from northern China) that recruit students from the whole country including international students. We selected sample that presents the university structure in terms of subjects, student number and studying years. This sampling strategy brought limitations: the sample employed in this study was only from two renowned universities and students from health sciences were over-represented. Therefore, the results may not be transferable to all university students specially studying at regional or local universities in China. However, it was beyond the researchers’ capacity to select representative sample of university students for a huge country like China that reveals remarkable economic and developmental differences among regions. Although we found statistical difference between OCs and NOCs in perceived stress and studying-related life satisfaction in Chinese subsample, we are cautious with the results due to the relatively small score difference between the Chinese NOCs and international NOCs.

The life satisfaction items we used were not very common compared with many studies in life satisfaction research and might complicate comparisons with results of those studies. However, as our study was the extension of the Cross National Student Health Study, our life satisfaction scale was adopted and only slightly extended from the valid scale, which was used in the European surveys conducted in several countries [28–30]. The small modifications were necessary to consider the specific Chinese conditions and–with focus on the studying-related aspects of university students–crucial to serve our study aims to compare Chinese with international students. Similar scales were also used in the USA and Canada to study students’ satisfaction in specific aspects of their learning experience [22, 45].

We found high correlations between perceived stress and studying-related life satisfaction (results not shown). It is possible that there is overlapping and interaction between the two measurements. Previous studies also reported that individuals with high life satisfaction and perceived less stress received more social support from their friends and family [8, 46]. Since life satisfaction is a significant component of well-being, dissatisfaction with life may be considered a symptom of stress [47]. It is difficult to imagine that persons experiencing high levels of stress would report high levels of life satisfaction.

Despite the fact that the majority of international students in our sample came from neighbouring countries, compared with their Chinese fellows the diversified cultural background and relatively small number in the international group may still have a certain impact on the study results. For example, Model R^2^s from Tables 4 and 5 are higher in international group as compared to Chinese group, but these are still insignificant.

Another limitation could be related to the categories of independent variables. For instance, small cities are defined in the Chinese context as cities at or lower than county level, they can mean differently for international students. Furthermore, it is important to point out that the international OC parents, who voluntarily choose to have only one child, they may practice a different style of upbringing compared with NOC parents under the OCP [11]. These factors may cause differences between the Chinese and international OCs in personality traits, which presumably may influence their perception of stress and life satisfaction. Given the cross-sectional character of our study, conclusions allow only associations and not causations. Finally, due to the distribution of the socio-economic characteristics, we dichotomized ordinal variables such as parents’ education and self-rated economic situation in analyses, this may cause some information loss.

## Conclusion

Since the implementation of the OCP, concerns have been raised over the alleged social deprivation and maladjustment of OCs in academia as well as in Chinese society more generally. Our study indicates that, compared with OCs, NOCs are disadvantaged at Chinese universities in perceived stress and studying-related life satisfaction. It has been reported that son preference and OCP were associated with female infant death in China [42]. Our findings imply that NOC men are also undertaking more pressure in this social context. Perceived stress and studying-related life satisfaction are individual phenomena, but they are also embedded in social, familial, and institutional contexts [8]. Therefore, comprehensive interventions for reducing stress, increasing life satisfaction, and promoting equality between OCs and NOCs among university students will require not only enhancing individual attitudes and competencies but also improving societal features and the university setting. Further exploration of the association between NOC status and increased stress as well as decreased studying-related life satisfaction in China is needed.

## Supporting Information

S1 File (PDF)
