# Supplementary material for: Only-Child Status in Relation to Perceived Stress and Studying-Related Life Satisfaction among University Students in China: A Comparison with International Students
Source: PLoS One. 2015 Dec 16;10(12):e0144947. doi: 10.1371/journal.pone.0144947 (PMC4686167; doi:10.1371/journal.pone.0144947)
Supplement: S1 File — (PDF) [file pone.0144947.s001.pdf]

STRESS AND HEALTH QUESTIONNAIRE

School of Public Health of PKU

School of Public Health of SYSU

School of Public Health of UBI

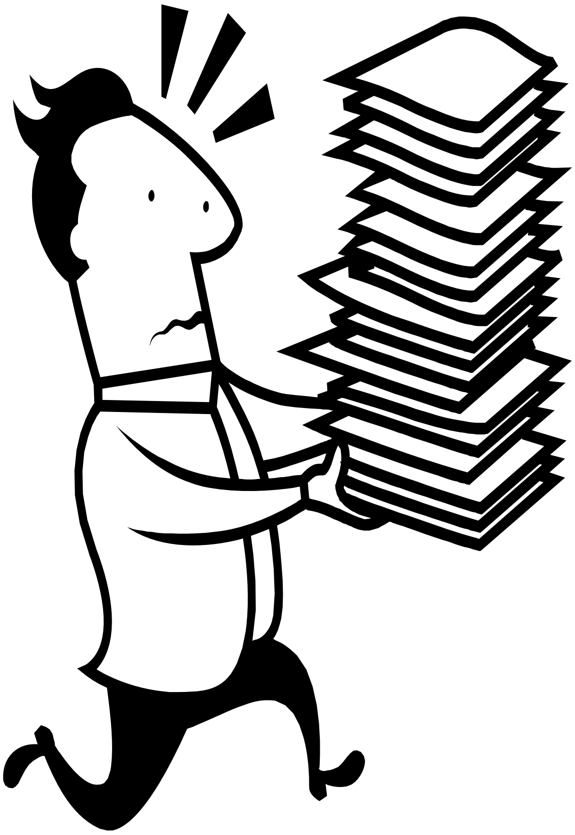

Dear students,

We would like to ask you to fill out the following questionnaire. The questionnaire deals with students' stress and health. The aim of this study is to develop a health promotion program based on the data collected which would be offered in future to students at PKU. Participation in the study is voluntary. By filling out this questionnaire you agree to it. At all times your identity will be kept confidential. The data will be used for scientific research purposes only. OR Your data will be ascertained anonymously, treated strictly in confidence and used exclusively for scientific purposes.

We would like to express our appreciation for your participation and cooperation in this project.

Tips for filling out the questionnaire:

Please answer all questions (including the same questions in different sections) honestly, openly and spontaneously. Since the questionnaire will be read by a scanner, please use a black or dark blue inked pen.

In judgmental statements please mark the field which corresponds to your point of view the most/the closest.

Example:

1. How satisfied do you feel today?

Not at all	A little	Somewhat	Quite satisfied	Satisfied	Very satisfied
☐	☐	☐	☐	☐	☑

Written fill-ins please write in block and capital letters in the given field. Keyword-answers are fully sufficient.

Example:

Other: 5000 Yuan/month.

In case there questions occur, please contact Mrs. Janet Chu via e-mail:

janet_junqing.chu@uni-bielefeld.de

First we would like to ask you about your general health condition.

1. How would you describe your health condition in general?

Excellent	Very good	Good	Poor	The worst
☐	☐	☐	☐	☐

2. Compared with the past year how would you describe your health condition?

Now much better than a year ago	Now a bit better than a year ago	Almost the same as a year ago	Now a bit worse than a year ago	Now much worse than a year ago
☐	☐	☐	☐	☐

3. How well do you take care of your health?

Not at all	Quite little	Quite lot	A lot
☐	☐	☐	☐

4. **The following statements relate to your well being in the last two weeks. Please mark all statements which correspond to the way you felt in the course of the last two weeks.**

In the course of the last two weeks	The whole time	Usually	More than the half of the time	A little less than half of the time	Once in a while	Never
I was happy and in a good mood.	☐	☐	☐	☐	☐	☐
I felt calm and relaxed.	☐	☐	☐	☐	☐	☐
I was full of energy and felt active.	☐	☐	☐	☐	☐	☐
I felt fresh and relaxed when I woke up.	☐	☐	☐	☐	☐	☐
My day was full of things which interested me.	☐	☐	☐	☐	☐	☐

The following part refers to different areas of your health.

5. Did you visit any doctor in the course of the last six months?

☐ No ☐ Yes; how often? ☐☐ What were the reasons ?......

6. Were you, in the course of the last twelve months, so ill that you had to stay in bed?

☐ No ☐ Yes; how many times? ☐☐ What was the illness?......

7. Do you take any medicine regularly?

☐ No ☐ Yes， what kind? ...., Why?...............

8.

Do you agree with the following statements?	I disagree	I agree to a little extent	I partially agree	I fully agree
Smoking should be forbidden in the university building/at university	☐	☐	☐	☐
Alcohol should not be sold at university.	☐	☐	☐	☐
The offer of healthy food at university is fully sufficient.	☐	☐	☐	☐
Chairs in most of class rooms are sufficiently comfortable.	☐	☐	☐	☐
Seats in most lecture halls are sufficiently comfortable	☐	☐	☐	☐
There is a nice atmosphere at university.	☐	☐	☐	☐

.

The following part deals with the degree of your satisfaction with various areas of your life.

9. To what extent are you satisfied With the following areas of your life?	Very unsatisfied					Very satisfied
1). Your studies in general	☐	☐	☐	☐	☐	☐
2). Your major and minor/subjects	☐	☐	☐	☐	☐	☐
3). Your grades at university	☐	☐	☐	☐	☐	☐
4). Your integration at university	☐	☐	☐	☐	☐	☐
5). Your job opportunities	☐	☐	☐	☐	☐	☐
6). Your flat	☐	☐	☐	☐	☐	☐
7). Your neighborhood	☐	☐	☐	☐	☐	☐
8). Place of study	☐	☐	☐	☐	☐	☐
9). Your free time	☐	☐	☐	☐	☐	☐
10). Your financial situation	☐	☐	☐	☐	☐	☐
11). Your friends	☐	☐	☐	☐	☐	☐
12). Relation with your parents/family	☐	☐	☐	☐	☐	☐
13).Your private life	☐	☐	☐	☐	☐	☐
14). Your health	☐	☐	☐	☐	☐	☐
15). Chinas as the country you study in –in general	☐	☐	☐	☐	☐	☐
16). Political situation in China	☐	☐	☐	☐	☐	☐
17). Economic situation in China	☐	☐	☐	☐	☐	☐

Considering once again your current situation:

18). How are you satisfied with your life in general?	☐	☐	☐	☐	☐	☐

The following part deals with discomforts/OR disorders/disturbances and various pressures in your life.

10. Which of the following discomforts/OR disorders/disturbances did you have in the course of the last year?

	Never	Seldom	Quite often	Very often
1). Stomach trouble/Heartburn	☐	☐	☐	☐
2). Low-back pain/Backache	☐	☐	☐	☐
3). Tiredness/Weariness	☐	☐	☐	☐

	Never	Seldom	Quite often	Very often
4). Breathing difficulties	☐	☐	☐	☐
5). Trembling hands	☐	☐	☐	☐
6). Tachycardia/Circulation disorder/Vertigo	☐	☐	☐	☐
7). Diarrhea	☐	☐	☐	☐
8). Constipation	☐	☐	☐	☐
9). Headaches	☐	☐	☐	☐
10). Sleep disorder/OR disturbance/insomnia	☐	☐	☐	☐
11). Nightmares	☐	☐	☐	☐
12). Concentration difficulties	☐	☐	☐	☐
13). Neck and arm ache	☐	☐	☐	☐
14). Abdomen disorder/OR Disturbance	☐	☐	☐	☐
15). Mood swings	☐	☐	☐	☐
16). Trembling	☐	☐	☐	☐
17). Depressive mood	☐	☐	☐	☐
18). Speech disorder	☐	☐	☐	☐
19. Weight gain/ Loss of weight	☐	☐	☐	☐
20. Lack of appetite	☐	☐	☐	☐
21. Nervousness/Anxiety	☐	☐	☐	☐
22). Fear/Phobia	☐	☐	☐	☐
23). Others	☐	☐	☐	☐

11.1. **To what extent do you feel impaired by the following?**

	Not at all					Very strongly
1). Studies in general	☐	☐	☐	☐	☐	☐
2). Exams, research papers, Presentations	☐	☐	☐	☐	☐	☐
3). Lack of practical relevance of studies/OR studies not oriented towards practical professions	☐	☐	☐	☐	☐	☐
4). Anonymity at university	☐	☐	☐	☐	☐	☐
5). Bad job prospects	☐	☐	☐	☐	☐	☐
6). Problems with parents	☐	☐	☐	☐	☐	☐

	Not at all					Very strongly
7). Problems with fellow students	☐	☐	☐	☐	☐	☐
8). Problems with friends	☐	☐	☐	☐	☐	☐
9). Private life	☐	☐	☐	☐	☐	☐
10). Flat/Living conditions	☐	☐	☐	☐	☐	☐
11). Problems with health	☐	☐	☐	☐	☐	☐
12). Financial situation	☐	☐	☐	☐	☐	☐
13). Workload of job/ pressures of working alongside	☐	☐	☐	☐	☐	☐
14). Isolation at university	☐	☐	☐	☐	☐	☐
15). Isolation in general	☐	☐	☐	☐	☐	☐
16). Lack of time for studies	☐	☐	☐	☐	☐	☐
17). Bad working conditions	☐	☐	☐	☐	☐	☐
18). Other	☐	☐	☐	☐	☐	☐

Considering once again your current situation:

19). To what extent do you feel impaired in general?	☐	☐	☐	☐	☐	☐

11.2 The following part refers to perceptions and thoughts in particular situations from your everyday life.

1). Do you have an impression that you are in an unusual situation and do not know what you should do/how to behave?

Very often — Very seldom or never

☐ ☐ ☐ ☐ ☐ ☐ ☐

2). When you think about your life it is often that...

You feel how beautiful it is to live — You ask yourself what for you live at all

☐ ☐ ☐ ☐ ☐ ☐ ☐

3). Things you do every day are for you ...

A source of great pleasure and satisfaction — A source of pain and boredom

☐ ☐ ☐ ☐ ☐ ☐ ☐

4). How often are your thoughts chaotic/OR messy?

Very often — Very seldom or never

☐ ☐ ☐ ☐ ☐ ☐ ☐

5). If you do something that makes you feel good ...

Then you will without doubt constantly feel so good — Then surely something will happen that will damage this

☐ ☐ ☐ ☐ ☐ ☐ ☐

6). You expect that your future life will have...

No clear goals or purposes at all — Very clear goals and purposes

☐ ☐ ☐ ☐ ☐ ☐ ☐

7). Many people – even those who have a strong character– feel like sad losers in certain situations. How often did you feel that way in the past?

Very often Seldom or never

☐ ☐ ☐ ☐ ☐ ☐ ☐

8). If you consider difficulties you may face in your life when doing important things, you have an impression that ...

You will successfully overcome the difficulties You will fail in overcoming the difficulties

☐ ☐ ☐ ☐ ☐ ☐ ☐

9). How often do you have an impression that things you do in your everyday life have little meaning?

Very often Very seldom or never

☐ ☐ ☐ ☐ ☐ ☐ ☐

11.3. Feelings and thoughts

The following questions deal with your feelings and thoughts in the course of the last four weeks. Please rate/OR estimate the frequency of particular impressions and feelings. Please answer each question even if it appears similar to other questions. If some events or changes, asked in the questionnaire, did not happen in the course of the last four weeks please rate/estimate even though how you would have felt. Please fill out the questionnaire uninterruptedly by marking on scale fields which correspond to your point of view the most/OR the closest.

In the course of the last four weeks.....

	Never				Very often
1). How often have you been upset because something unexpected happened in your life?	☐	☐	☐	☐	☐
2). How often have you felt that the most important things in your life were out of your control?	☐	☐	☐	☐	☐
3). How often have you felt nervous and tense?	☐	☐	☐	☐	☐
4). How often have you succeeded in dealing with unpleasant events?	☐	☐	☐	☐	☐
5). How often have you had an impression that you were able to deal with important changes in your life?	☐	☐	☐	☐	☐
6). How often have you felt sure that you were able to deal with your personal problems well enough?	☐	☐	☐	☐	☐
7). How often have you had an impression that things in your life developed as you planned?	☐	☐	☐	☐	☐
8). How often have you had an impression that you did not meet everyday demands?	☐	☐	☐	☐	☐
9). How often have you succeeded in getting rid of vexations/OR nuisances from your way?	☐	☐	☐	☐	☐
10). How often have you had an impression that you were at the top?	☐	☐	☐	☐	☐
11). How often have you felt angry that things happened which were out of your control?	☐	☐	☐	☐	☐
12). How often have you noticed that you thought about things which you had to complete?	☐	☐	☐	☐	☐
13). How often have you felt to be able to spend your time freely?	☐	☐	☐	☐	☐
14). How often have you had an impression that difficulties overwhelmed you so much that you were not able to accomplish them?	☐	☐	☐	☐	☐

11.4. The following questions refer to relationship with important people, such as to partner, family members, friends, acquaintances, colleagues/OR fellow students and neighbors.

Do you agree with the following statements?	**No**				**Yes**
1). I easily find someone who can take care of my flat (plants, domestic animals, mail, etc.) when I am away.	☐	☐	☐	☐	☐
2). There are people who take me as I am without any restrictions.	☐	☐	☐	☐	☐
3). I have experienced a lot of understanding and security from others.	☐	☐	☐	☐	☐
4). I have a reliable person upon whose help I can always depend.	☐	☐	☐	☐	☐
5). If it is necessary I can always borrow anything from friends or neighbors.	☐	☐	☐	☐	☐
6). I have friends/relatives who always spare the time to listen to me.	☐	☐	☐	☐	☐
7). I know many people with whom I can do something.	☐	☐	☐	☐	☐
8). I have friends/relatives who can hug me.	☐	☐	☐	☐	☐
9). If I am ill, I can ask my friends without any hesitation to do things for me such as shopping.	☐	☐	☐	☐	☐
10). If I am very depressed I know to whom I can go.	☐	☐	☐	☐	☐
11). There are people who share happiness and sorrow with me	☐	☐	☐	☐	☐
12). With some friends I can be jolly at times.	☐	☐	☐	☐	☐
13). I have a group of reliable friends in whose presence I can feel good without any restrictions.	☐	☐	☐	☐	☐
14). There is a group of people (a group of Closest friends, clique) to whom I belong and with whom I spend most of my time.	☐	☐	☐	☐	☐

12. How many people do you know – including your family – who support you whenever you feel depressed?

None	One person	Two - three persons	More than three persons
☐	☐	☐	☐

13. Are you on the whole satisfied with support you get in such cases?

Very satisfied				Very unsatisfied
☐	☐	☐	☐	☐

Quality of and attitude to life

14. If you consider the quality of your life: How did the things go in the last four weeks?

Very badly	Badly	So So	Quite well	Very well
☐	☐	☐	☐	☐

14.1 **The following questions refer to your present attitude to life. In every question please indicate how frequently you have experienced the following mood and perception.**

	Never					Almost always
1). I am sad.	☐	☐	☐	☐	☐	☐
2). I look into the future in a discouraged way.	☐	☐	☐	☐	☐	☐
3). I feel like a goof.	☐	☐	☐	☐	☐	☐
4). It is difficult to enjoy anything.	☐	☐	☐	☐	☐	☐
5). I feel guilty.	☐	☐	☐	☐	☐	☐
6). I feel as if I am being punished.	☐	☐	☐	☐	☐	☐
7). I am disappointed with/of myself	☐	☐	☐	☐	☐	☐
8). I point out mistakes to myself.	☐	☐	☐	☐	☐	☐
9). I consider hurting myself.	☐	☐	☐	☐	☐	☐
10). I cry	☐	☐	☐	☐	☐	☐
11). I feel nervous, angry and annoyed.	☐	☐	☐	☐	☐	☐
12). I do not care about other people.	☐	☐	☐	☐	☐	☐
13). I put off making decisions.	☐	☐	☐	☐	☐	☐
14). I care about my outer appearance.	☐	☐	☐	☐	☐	☐
15). I have to force myself to every task.	☐	☐	☐	☐	☐	☐
16). I cannot sleep well.	☐	☐	☐	☐	☐	☐
17). I am tired and dull.	☐	☐	☐	☐	☐	☐
18). I do not have appetite.	☐	☐	☐	☐	☐	☐
19). I am afraid of my health.	☐	☐	☐	☐	☐	☐
20). I do not care about sex.	☐	☐	☐	☐	☐	☐

14.2 **Now we would like to ask you a couple of questions with regard to how you perceive yourself.**

In how much do you agree with the following statements?	I disagree	I agree partly	I agree	I fully agree
1) If I face resistance, I find means to establish/OR assert myself/to become accepted.	☐	☐	☐	☐
2) I succeed in resolving difficult problems if I do my best.	☐	☐	☐	☐
3) I have no difficulties in realizing my intentions and aims.	☐	☐	☐	☐
4). In unexpected situations I know how to behave.	☐	☐	☐	☐
5). Even with surprising events I believe I can deal with them.	☐	☐	☐	☐
6). I approach difficulties calmly because I believe in my skills.	☐	☐	☐	☐
7). Whatever happens, I will be ok.	☐	☐	☐	☐

8). For each problem I can find a solution. ☐ ☐ ☐ ☐

9). Whenever I experience something new, I know how to deal with it. ☐ ☐ ☐ ☐

10). If I face a problem, I can deal with it on my own. ☐ ☐ ☐ ☐

Wishes and aspirations

You have three wishes with regard to destiny – what do you wish? Choose three possibilities and put them in order.

Freedom in the world, more understanding among people, Freedom, security for all, Health, Happy family, Privacy, Holidays, Lots of money, Financial security, Success, Happiness, True love, Pleasure/enjoyment, True friendship, Wisdom, Other. 1. ______________, 2. _______________, 3. _______________.

15. If you could, would you like to leave your current place of residency and move somewhere else?

Not at all — Very strongly

☐ ☐ ☐ ☐ ☐

16. What would be the decisive reasons for you leaving your current residency?

________________________; ______________________________.

17. In which countries could you imagine living for a longer period of time?_________

The following questions refer to your nourishment.

18. How often do you consume the following foods?

	Many times per day	Daily	Many times per week	1-4 times per month	never
1. Sweets (chocolate, sweets, etc.)	☐	☐	☐	☐	☐
2. Cakes/cookies	☐	☐	☐	☐	☐
3. Snacks (chips, peanuts, etc.)	☐	☐	☐	☐	☐
4. Fast food/canned food (hamburger, French fries, instant noodles etc.)	☐	☐	☐	☐	☐
5. Fresh fruits	☐	☐	☐	☐	☐
6. Salad/raw vegetables	☐	☐	☐	☐	☐
7. Cooked vegetables	☐	☐	☐	☐	☐
8. Lemonade/soft drinks	☐	☐	☐	☐	☐
9. Meat/sausage products					

	Many times per day	Daily	Many times per week	1-4 times per month	never
10. Fish/sea food	☐	☐	☐	☐	☐
11. Milk/milk products	☐	☐	☐	☐	☐
12. Cereal/cereal products (whole wheat bread, oat flakes, etc.)	☐	☐	☐	☐	☐

19. How important is for you to nourish healthy? Very important — Unimportant at all

☐ ☐ ☐ ☐ ☐

20. What are the reasons for your unhealthy eating habits?

	I agree				I fully disagree
Lack of appetite	☐	☐	☐	☐	☐
Too little time	☐	☐	☐	☐	☐
Lack of time	☐	☐	☐	☐	☐

Other: ....

21. How often do you spend time on physical activity (sport, physical work, etc.) in a normal week that takes you at least 20 minutes, makes you breathe deeper and significantly quickens your pulse?

Less than once	Once or twice	At least three times
☐	☐	☐

22. What is your height? ☐☐☐ cm
23. What is your weight? ☐☐kg
24. How satisfied are you with your current weight?

Very satisfied	Quite satisfied	Quite unsatisfied	Unsatisfied
☐	☐	☐	☐

25. In your opinion you are:

Much too thin	A bit too thin	Just right	A bit too fat	Much too fat
☐	☐	☐	☐	☐

The following part relates to smoking, drugs and alcohol:

26. How often in the course of the last three months did you smoke? (cigarettes, pipe, small cigars, cigars)
☐daily ☐occasionally ☐never （**no smokers skip 27，28**）
27. In case you smoke daily: How many cigarettes do you smoke daily on average?☐☐
28. Did you try to give up smoking in the course of the last 12 months? Yes ☐ No ☐
29. Have you ever tried or taken drugs?☐Yes, regularly☐Yes, only tried☐No, never tried (**never tried skip30**)
30. If so, which one? (hashish, cocaine, heroin, crack, LSD, ecstasy?)
31. How often did you drink alcohol, e.g. beer, in the course of the last three months?

Many times per day	Every day	Many times per week	Once per week	Less frequent than once a week	Never
☐	☐	☐	☐	☐	☐ **(No drinkers skip 32)**

32. Do you agree with the following statements?

	I agree	disagree
I have drunk alcohol in the morning as my first drink to psychically balance myself and to get rid of hangover.	☐	☐
I have already had the impression that I need to reduce my alcohol consumption.	☐	☐
Someone has made me angry by criticizing my drinking.	☐	☐
I have felt bad and guilty because of my drinking.	☐	☐

A couple of questions with regard to your studies.

33. Which semester are you currently in? in☐☐semester
34. What do you study? …………
35. How important is it for you to have good grades/OR to achieve well at university?

Very important	Quite important	Not very important	Unimportant
☐	☐	☐	☐

36. How do you rate your performance in comparison with the average performance of your fellow students?

Much better	Better	The same	Worse	Much worse
☐	☐	☐	☐	☐

37. Do you have any particular plans for the time after your studies?

No☐ Yes☐, what............................

38. If so, how certain are you that you will be able to realize your plans?

Certain	Quite certain	Not very certain	Uncertain
☐	☐	☐	☐

Now some personal details

39. How old are you? ☐☐years

40. What is you sex? ☐female ☐male

41. Where were you born? ☐Countryside ☐Small city ☐Medium size/big city

42. Are you an Only-child? ☐Yes ☐ No

43. Are you a religious person? ☐ No ☐ yes, if yes, which religion____

44. How strongly can you agree with the following statement: “my belief has the biggest influence on my life”?

fully agree	agree	undecided	disagree	fully disagree
☐	☐	☐	☐	☐

45. How do you estimate the amount of money you have?

Fully sufficient	Sufficient	Less sufficient	Fully insufficient
☐	☐	☐	☐

46. In what way do you mostly finance your studies? (more marks possible)
    - ☐ parents’ support
    - ☐ student loan
    - ☐ occupation during semester
    - ☐ occupation during breaks
    - ☐ scholarship
    - ☐ other: ....

47. Your yearly expense equals ___% of your entire family annual income.

48. What education degree have or did have your parents?

	Mother	Father
No education degree	☐	☐
Elementary school/secondary school	☐	☐
Junior/vocational school	☐	☐
Advanced technical college/Higher education entrance qualification	☐	☐

49. Do you have a boyfriend/a girlfriend? ☐No ☐Yes, if yes, how long have you been together, for ...... years or for ...... months.

50. Are you a foreign student? ☐No ☐Yes, if yes, from which country__________.

Thank you for your cooperation!
